# Anti-inflammatory effects of powdered product of Bu Yang Huan Wu decoction: Possible role in protecting against Transient Focal Cerebral Ischemia

**DOI:** 10.7150/ijms.46581

**Published:** 2020-07-11

**Authors:** Kuan-Yu Chen, Kuo-Chen Wu, Dueng-Yuan Hueng, Kuo-Feng Huang, Cheng-Yoong Pang

**Affiliations:** 1 Department of Surgery, New Taipei City Hospital, New Taipei city, Taiwan.; 2 Institute of Medical Sciences, Tzu Chi University, Hualien city, Taiwan.; 3 School of Pharmacy, National Taiwan University, Taipei, Taiwan.; 4 Department of Neurosurgery, Tri-Service General Hospital, National Defense Medical Center, Taipei, Taiwan.; 5 School of Medicine, Buddhist Tzu Chi University, Hualien, Taiwan.; 6 Division of Neurosurgery, Department of Surgery, Taipei Tzu Chi Medical Hospital, Buddhist Tzu Chi Medical Foundation, New Taipei City, Taiwan.; 7 Department of Medical Research, Buddhist Tzu Chi General Hospital, Hualien city, Taiwan.

**Keywords:** Stroke, Ischemic/reperfusion, inflammation, Bu Yang Huan Wu decoction

## Abstract

Bu Yang Huan Wu decoction (BYHW) is a traditional Chinese medicine (TCM) that consists of several herbs and has been used in patients with ischemic stroke for centuries. Although powdered formula of BYHW has widely been prescribed in clinic nowadays, evidence-based effectiveness and mechanism of action of BYHW powdered product in stroke remain to be characterized. Adult male Sprague-Dawley rats were subjected to middle cerebral artery occlusion (MCAO) for 90 min followed by reperfusion for 24 h (ischemia/reperfusion; I/R) or sham surgery. After I/R, the rats were then given low dose (0.5 g/kg) and high dose (2.5 g/kg) of BYHW or vehicle by oral gavage twice a day for seven consecutive days. The results showed that I/R induced obvious cerebral infarction and neurobehavioral defects, in parallel with histological aberrations and extensive signaling of proinflammatory cytokines, including tumor necrosis factor (TNF-α) and interleukin-6 (IL-6), in the stroke model. Post-I/R treatment with BYHW powdered product significantly reduced the infarct area and ameliorated neurofunctional defects in a dose-dependent manner. The dose dependence was associated with TNF-α downregulation and interleukin-10 (IL-10) induction. In summary, the present findings demonstrated that BYHW powdered product exhibited therapeutic efficacy for experimental stroke and a higher dose treatment may strengthen the effectiveness via inflammatory modulation.

## Introduction

Ischemic stroke is a leading cause of death worldwide. The treatments for acute ischemia stroke are to provide intravenous recombinant tissue plasminogen activator (tPA) or angiographic thrombolysis that can restore the blood stream 1. However, duo to its limited therapeutic time window, contraindications, and potential reperfusion injury, the rate of patients with ischemic stroke receiving tPA therapy is low 2. Although extensive studies have demonstrated that the mechanisms underlying neuronal death in ischemic stroke are associated with glutamate-mediated excitotoxic damage, blood-brain barrier injury, oxidative stress, and inflammation 3, other effective therapeutic options for the treatment of ischemic stroke are still limited.

Traditional Chinese medicines (TCMs), including a classic formula Bu Yang Huan Wu decoction (BYHW), have long been used in patients with brain disorders including cerebrovascular diseases. BYHW is composed of Huangqi (Radix Astragali seu Hedysari), Danggui (Radix Angelica sinensis), Chishao (Radix Paeoniae Rubra), Chuanxiong (Rhizoma Ligustici Chuanxiong), Honghua (Flos Carthami), Taoren (Semen Persicae) and Dilong (Pheretima). The chief drug of BYHW, Radix Astragali, contains flavonoids and saponins that are suggested to be responsible for its major pharmacological activities. After oral administration of BYHW, astragaloside I, astragaloside II, astragaloside IV, formononetin, ononin, calycosin, calycosin-7-O-β-d-glucoside, ligustilide and paeoniflorin can be detected in plasma of rats 4. Consistent with the bioactivities of these compounds, accumulating evidence shows that BYHW can ameliorate multiple pathological pathways of ischemic stroke, such as oxidative stress, inflammation, and apoptosis 5. Among cerebral ischemic models, middle cerebral artery (MCA) occlusion (MCAO) is widely used to investigate the therapeutic potential of TCMs 6. In rats with MCAO, BYHW is found to inhibit elevation of excitatory amino acids and normalized the increase of metabotropic glutamic acid receptor-1 expression 7. In addition, BYHW and its herbal components also induce the expression of angiogenesis-related proteins 8 and inhibit upregulation of toll-like receptor 4 after cerebral ischemic injury 9. Although a recent report using array analysis suggested that BYHW affects inflammation-related gene expression in the MCAO brains 9, it is still not well-understood whether and how regulation of inflammation is involved in the protection of BYHW against MCAO-induced infarction and neurobehavioral deficits.

Nowadays, many traditional herbal decoctions, including BYHW, are processed to be powdered products for the convenient use by patients with ischemic stroke. However, the evidence-based therapeutic efficacy of BYHW powdered products in ischemic stroke is lacking. Therefore, the present study aimed to investigate the therapeutic effects of different doses of BYHW powdered product in ischemic stroke rat model and to evaluate its underlying mechanism via regulating inflammation.

## Materials and Methods

### Experimental animals

Male Sprague-Dawley rats (six week old), weighting 250 ~ 300g, were purchased from BioLasco Co., Ltd. (Taipei, Taiwan). All rats were housed in an animal center with specific pathogen free condition and with a 12-h light-dark cycle for 7 days to acclimate to the environment prior to experimentation. The animals had free access to standard rodent diet and water at all times. The experimental protocol used in this study was reviewed and approved by the Ethics Committee for Animal Experimentation of Taipei Tzu Chi Hospital (approval no. 107-IACUC-002).

### Focal cerebral ischemia and reperfusion (I/R) surgery

Focal brain ischemia model was established by MCAO, as described previously 10. Briefly, rats were anesthetized by a mixture of Zoletil 50 (50 mg/kg) and xylazine (10 mg/kg) intraperitoneally. The right common carotid artery was ligated to reduce the cerebral blood flow. Afterwards, rats were fixed on a stereotaxic instrument and the right MCA was tied with a 10/0 nylon line. After 90 min of occlusion, the nylon line was gently removed to allow reperfusion for 24 h. At initiation of occlusion and reperfusion, blood flow of the MCA was verified visually under a microscope. In sham-operated rats, the incision was sutured but MCA was not occluded. After the surgery was performed, each rat was housed individually to avoid wound dehiscence.

### Drug intervention

Rats were randomly divided into four groups: sham-operated (negative control), MCAO (ischemia)/reperfusion (I/R), I/R + low-dose BYHW (0.5 g/kg/bid), and I/R + high-dose BYHW (2.5 g/kg/bid). The powdered product of BYHW was obtained from Sun-Ten Pharmaceutical Co. Ltd. (New Taipei City, Taiwan) and manufactured under PIC/S GMP guidelines. The components of the powdered BYHW were listed in Table 1. After I/R, rats were received BYHW or vehicle for seven consecutive days. Rats in sham-operated group were orally administered vehicle (saline). At 24 h after the last administration of saline or BYHW, rats were sacrificed for histological and biochemical studies. Neurobehavioral tests were performed at day 1, day 7, and day 14 after the onset of I/R. Euthanasia was performed by introduction of carbon dioxide into a cage at a sufficient rate.

### Neurobehavioral assessment

Neurobehavioral performance was evaluated using corner test and modified neurological severity score (mNSS). The corner assessment was performed as described previously 11. The mNSS included motor test, sensory test, beam balance test, and reflexes absent and abnormal movement test as described previously 12.

### Examination of cerebral infarct volume

The cerebral infarct area was examined by 2,3,5-triphenyltetrazolium chloride (TTC) staining as described previously 13. Rats were perfused with ice-cold normal saline via the left ventricle. The brains were carefully removed and sectioned at the thickness of 2 mm. The brain slices were stained in 2,3,5-triphenyltetrazolium chloride (TTC) solution (2%) for 30 min at 37 °C and then immersed in 10% formalin overnight. The infarct areas were calculated with Image-J (v1.46r, Wayne Rasband, National Institutes of Health, USA). The percentage infarction was calculated as (infarct area/area of whole sections) × 100%.

### Hematoxylin and eosin (HE) staining

For HE staining, animals were perfused transcardially with 200 mL normal saline and then with 300 mL ice-cold 4% paraformaldehyde in 0.1M phosphate buffer (pH 7.4). The brains were embedded in paraffin and sequentially sectioned into slices of 3 μm thickness. After rehydration with serial alcohol, the sections were stained with hematoxylin and eosin following standard procedures. The morphology was examined using a light microscopy at 40× and 200× magnification. The HE staining was used to evaluate the degree of cell necrosis, cell connection, and cell infiltration.

### Immunohistochemistry

The brain sections were prepared according to the protocol described above. The brain sections were blocked with 3% H_2_O_2_ for 15 min and subjected to normal goat serum for 15 min. Then, the sections were incubated with rabbit polyclonal anti-tumor necrosis factor-α (TNF-α) (1:500; Abcam, Cambridge, UK) and interleukin-6 (IL-6) (1:500; Abcam, Cambridge, UK) overnight at 4 °C. After washing in PBS (pH 7.4) three times for 3 min, the sections were incubated with corresponding horseradish peroxidase (HRP) conjugated secondary antibodies for 30 min at room temperature. Finally, the slides were washed again in PBS and stained with 0.01% 3, 3-diaminobenzidine tetrahydrochloride (DAB) for approximately 1 min. A dark brown color was regarded as a positive staining signal. The positive area viewed using a light microscope. The average integral optical density (AIOD) was calculated as positive area ∗ OD/total area.

### Enzyme-linked immunosorbent assay

The levels of TNF-α, transforming growth factor-β (TGF-β), IL-6, interleukin-10 (IL-10), malondialdehyde (MDA) in brain tissues were measured by enzyme-linked immunosorbent assay (ELISA) as described by the manufacturer's instruction. In brief, rat brains in the experimental groups were quickly removed from the skull and homogenized in ice-cold buffer. After centrifugation, the supernatants were collected and reacted with the commercial ELISA kits (R&D Systems Inc., Minneapolis, MN, USA). The intensity of the enzyme reaction was examined at 450 nm using a microplate reader.

### Statistical analysis

The data were expressed as the mean ± standard deviation (SD). All statistical analyses were performed using SPSS 17.0 software (Chicago, IL, USA). The statistical differences between groups were analyzed using one-way ANOVA, followed by Least Significant Difference (LSD) test. The significance level was taken as P < 0.05.

## Results

### BYHW powdered product ameliorated I/R-induced infarct damage in the cerebral cortex of rats

**Figure 1:**
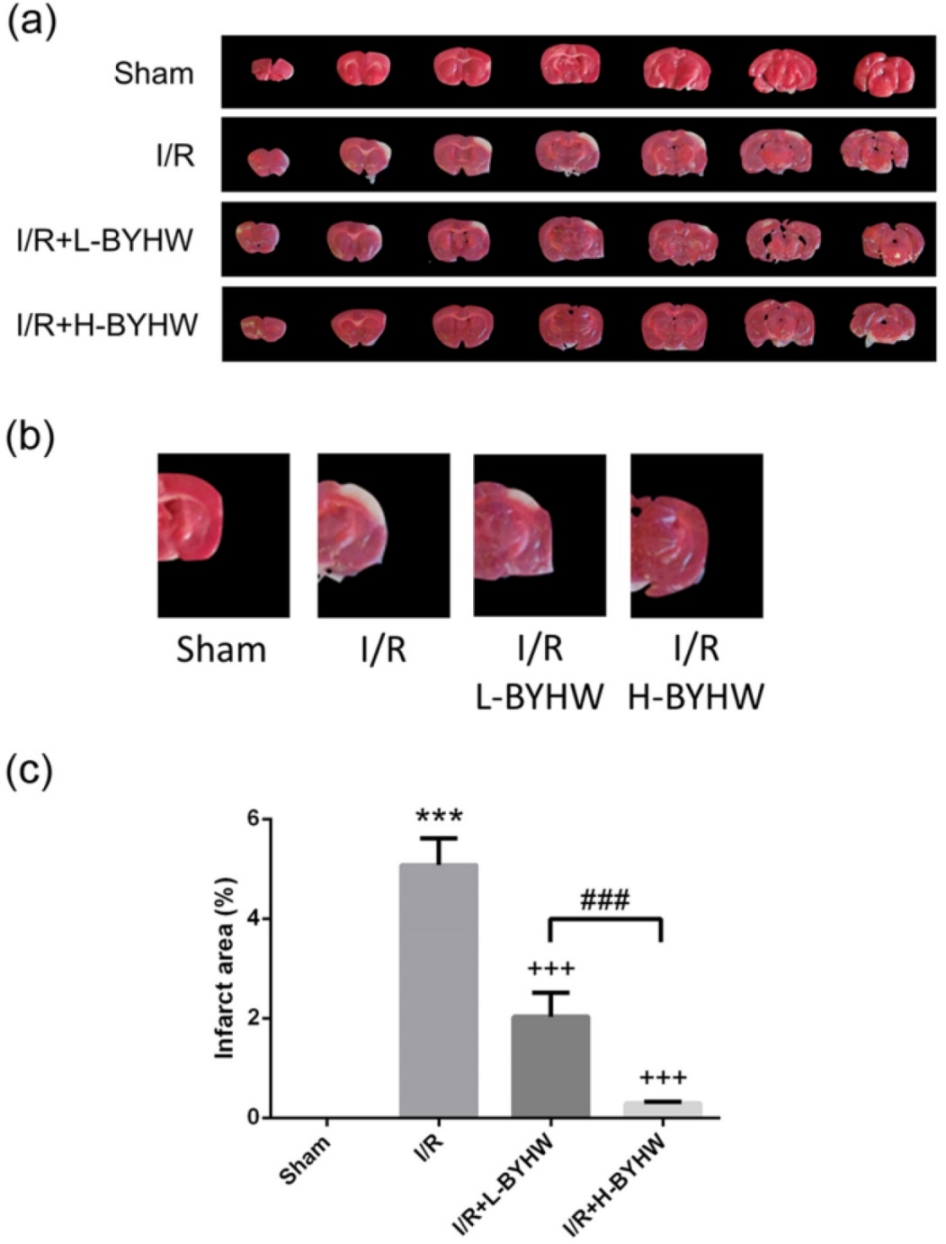
Powdered product of BYHW ameliorates I/R-induced infarct damage in the cerebral cortex of rats. (**a**) Representative 2,3,5-triphenyltetrazolium chloride (TTC)-stained coronal cortical sections from sham-operated rats and MCAO/reperfusion (I/R) rats receiving vehicle, low-dose or high-dose powdered BYHW. (**b**) Higher magnification images of the fourth sections of all groups are presented. (**c**) The area of infarcted brain tissue at 7 days after sham operation or I/R was estimated and expressed as a percentage of the whole section area. Data were presented as mean ± SD (n = 3 in each group). ***, P < 0.001 compared with sham-operated group; +++, P < 0.001 compared with I/R group; ###, P < 0.001 between groups treating with low-dose and high-dose powdered BYHW.

To evaluate I/R-induced cerebral infarction, TTC staining was performed in the brains obtained from rats receiving 90 min of MCAO and 24 h of reperfusion or sham surgery. Sham group underwent surgery procedure without I/R showing no infarct area, whereas the brain from I/R rats showed obvious infarct area in the cerebral cortex (5% of total area). The infarct area was slightly reduced on day 8 after MCAO onset in the cerebral cortex of rats treated with low dose of BYHW (2% of total area) and was apparently reduced in group treated with high dose BYHW (1% of total area) (Figure 1A-C; higher magnification of images shown in B). These data show that powdered BYHW has ability to reduce I/R-induced cerebral infarction in a dose-dependent manner.

### BYHW powdered product ameliorated I/R-induced histological alterations in the cerebral cortex of rats

**Figure 2:**
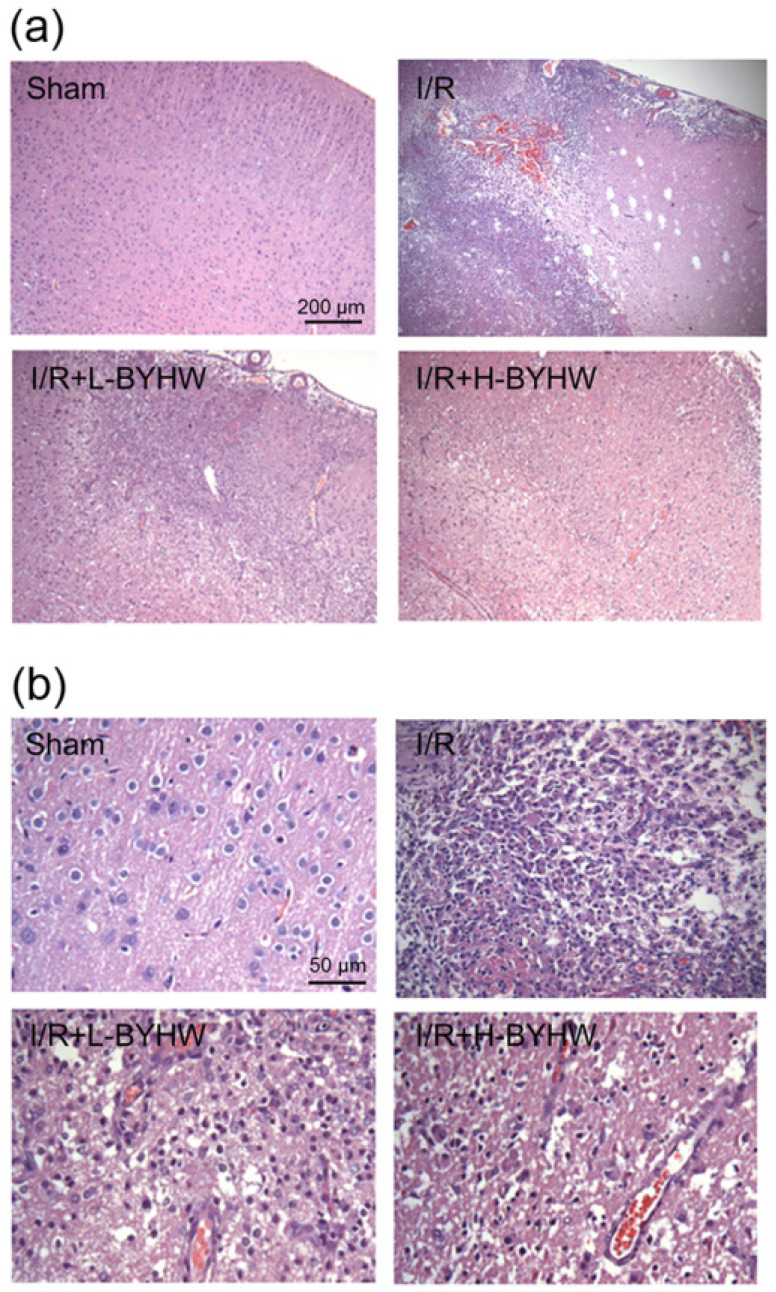
Powdered product of BYHW ameliorates I/R-induced histological alterations in the cerebral cortex of rats. (**a**) Representative results of Hematoxylin and eosin (HE) staining in cortical sections from sham-operated rats and MCAO/reperfusion (I/R) rats receiving vehicle, low-dose or high-dose powdered BYHW. (**b**) High magnification of HE staining. Scale bars: 200 µm in (a); 50 µm in (b).

Next, the effects of powdered BYHW on brain histopathological alterations in the ischemic penumbra were examined by HE staining. After I/R, numerous shrunken neurons with nuclei shrinkage and cell infiltration were distributed in the damaged brain tissue. In addition, microvascular disruption and cerebral bleeding were also seen in I/R group. Compared with I/R group, the neuronal damage and cell infiltration were shown to be ameliorated in I/R rats when BYHW was applied (Figure 2A). Higher magnification images were shown in Figure 2B.

### BYHW powdered product ameliorated I/R-induced neurobehavioral impairment in rats

**Figure 3:**
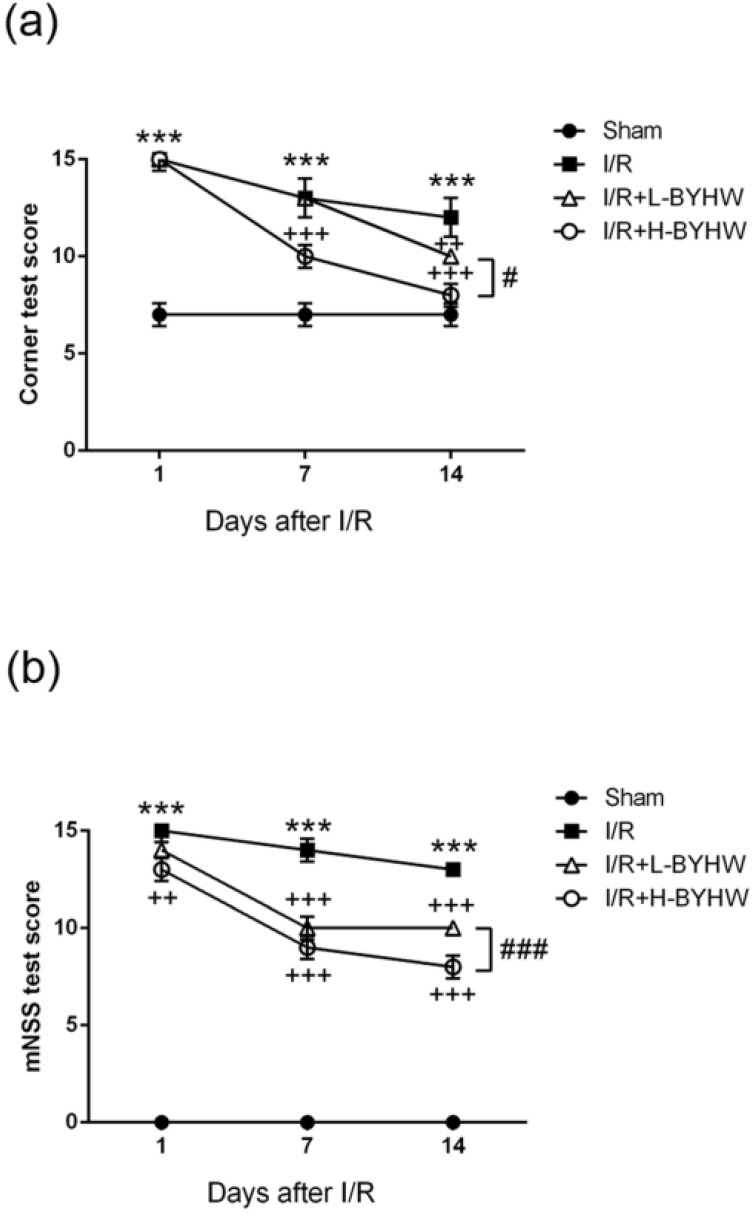
Powdered product of BYHW ameliorates I/R-induced neurobehavioral impairment in rats. (**a and b**) Corner test (a) and modified neurological severity scores (mNSS) (b) of sham-operated rats and MCAO/reperfusion (I/R) rats receiving vehicle, low-dose or high-dose powdered BYHW at 1, 7, and 14 days after sham operation and I/R. Data were presented as mean ± SD (n = 3 in each group). ***, P < 0.001 compared with sham-operated group; ++, P < 0.01 and +++, P < 0.001 compared with I/R group; #, P < 0.05 and ###, P < 0.001 between groups treating with low-dose and high-dose powdered BYHW.

Given that it has been demonstrated that BYHW has protective effects on I/R-induced histological changes, we next investigate whether BYHW treatment is beneficial for neurobehavioral defects in I/R rats. In sham group, we found that the score of corner test was 7 points, showing no neurobehavioral impairments due to surgical procedure. The score of corner test in I/R group was 15 points on the first day, 14 points on the seventh day and 13 points on the fourteenth day after reperfusion. While the score of corner test in low dose of BYHW-treated I/R rats was 15, 14, 11 points on 1st, 7th, and 14th days, respectively, the score in high dose of BYHW-treated group was 15, 10, 8 points on 1st, 7th, and 14th days, respectively (Figure 3A).

To further evaluate I/R-induced neurological deficits including motor, sensory, balance, and reflex, the mNSS was performed at post-reperfusion day 1, 7, and 14. The results showed that I/R operation significantly increased the score (15, 14, 13 points on 1st, 7th, and 14th days, respectively) compared with that of sham group (0, 0, 0 points on 1st, 7th, and 14th days, respectively). The score of mNSS was 14, 11, 10 points on 1st, 7th, and 14th days, respectively, in low-dose BYHW-treated I/R rats and the score was 13, 9, 8 points on 1st, 7th, and 14th days, respectively, in high-dose BYHW-treated group (Figure 3B). Our results demonstrate that BYHW powdered product has rescue effects against I/R-induced histological damage and neurobehavioral impairment in rats. The dose-dependent effects of BYHW on histological preservation and behavioral improvement are consistent with the results of evaluation of infarct area.

### BYHW powdered product reduced the expression of pro-inflammatory and increased anti-inflammatory cytokines in the cerebral cortex of rats

**Figure 4:**
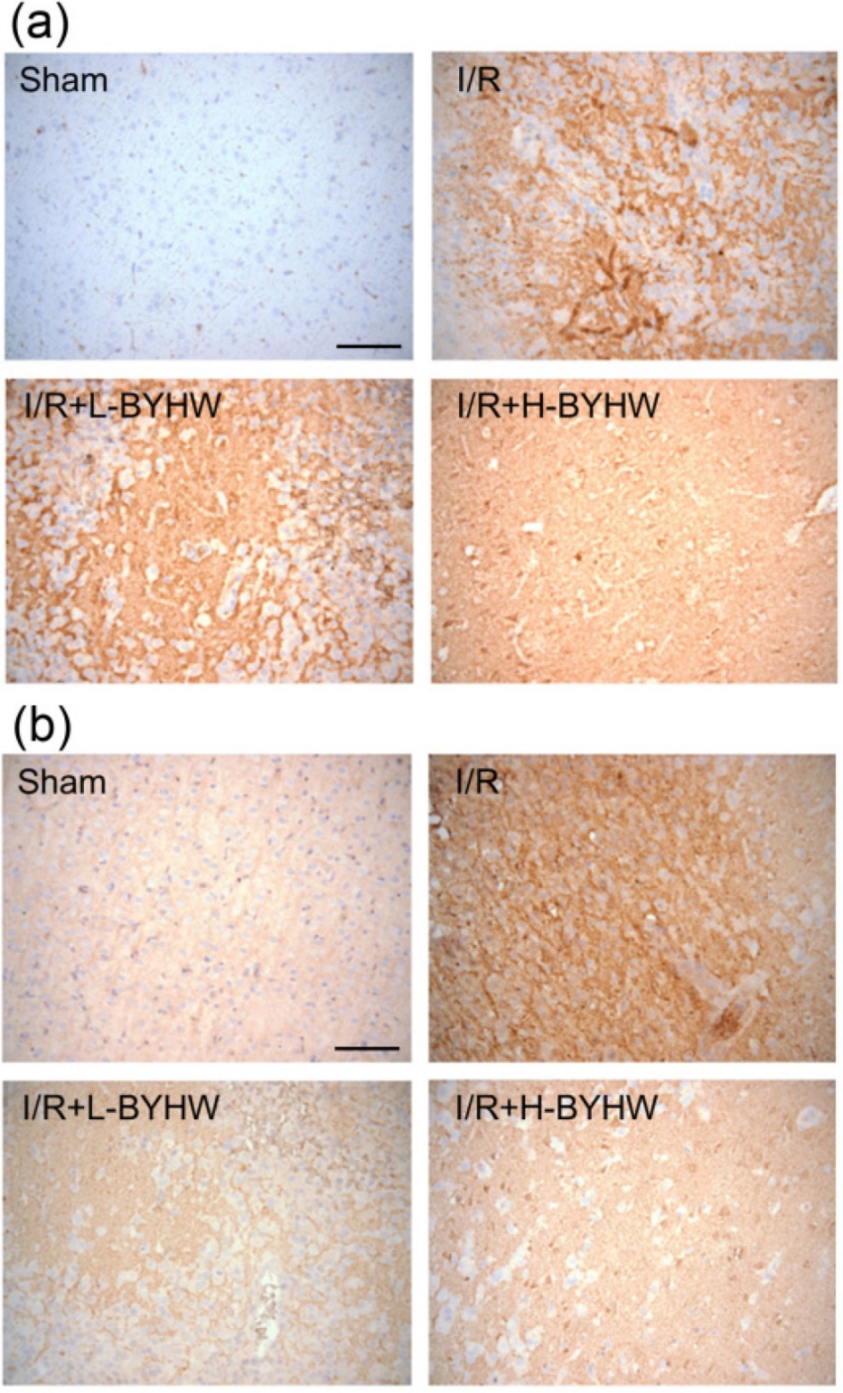
Powdered product of BYHW reduces the expression of TNF-α and IL-6 in the cerebral cortex of MCAO/reperfusion (I/R) rats. (**a and b**) Representative results of immunohistochemical staining (IHC) for TNF-α (a) and IL-6 (b) in cortical sections from sham-operated rats and I/R rats receiving vehicle, low-dose or high-dose powdered BYHW. Scale bars: 100 µm.

Inflammation has been implicated in the pathogenesis of cerebral ischemic injury 1. To investigate whether BYHW treatment downregulated the expression of major pro-inflammatory cytokines, immunohistochemistry for TNF-α and IL-6 was performed on the cortical slices obtained from sham-operated and I/R rats with or without BYHW treatment. The results of immunohistochemical staining showed that the expression of TNF-α and IL-6 were higher in the cerebral cortex of I/R rats compared with the sham group. Both low and high dose of BYHW treatments significantly reversed I/R-induced increase of TNF-α (Figure 4A) and IL-6 (Figure 4B).

**Figure 5:**
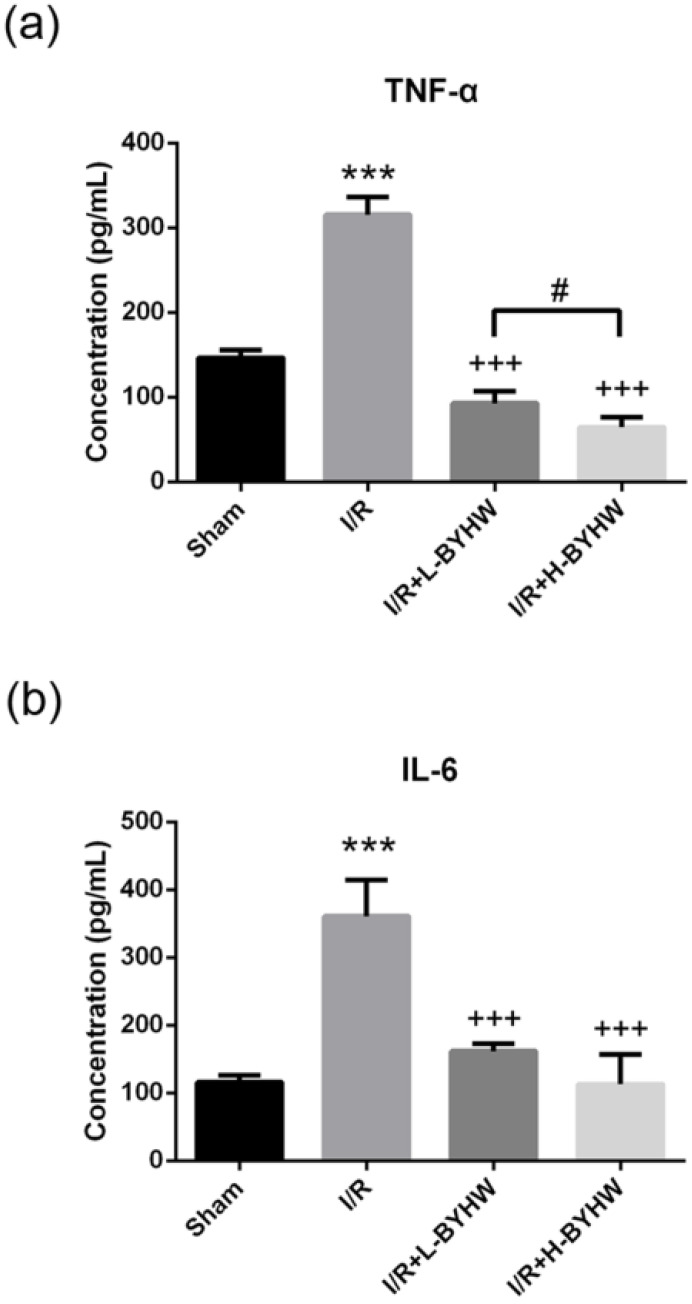
Powdered product of BYHW reduces the protein levels of TNF-α and IL-6 in the brain of MCAO/reperfusion (I/R) rats. (**a and b**) The protein levels of TNF-α (a) and IL-6 (b), as measured by ELISA, in the brains obtained from sham-operated rats and I/R rats receiving vehicle, low-dose or high-dose powdered BYHW at 7 days after sham operation or I/R. Data were presented as mean ± SD (n = 3 in each group). ***, P < 0.001 compared with sham-operated group; +++, P < 0.001 compared with I/R group; #, P < 0.05 between groups treating with low-dose and high-dose powdered BYHW.

**Figure 6:**
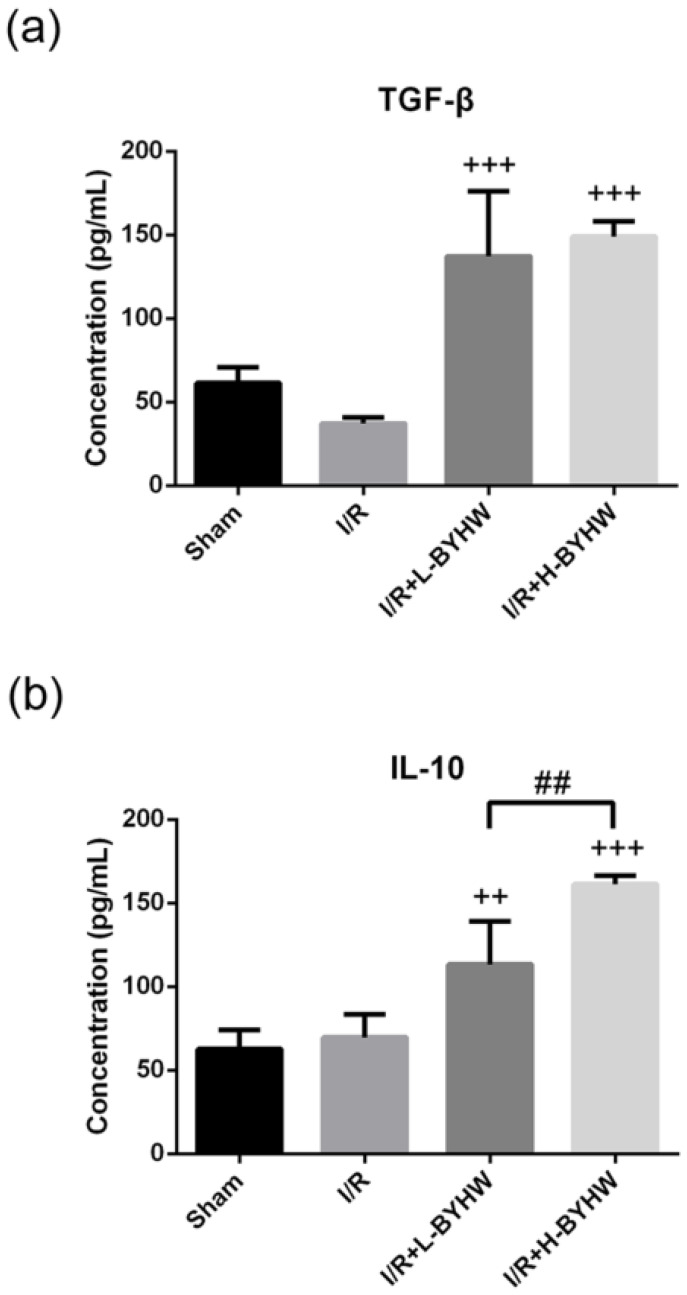
Powdered product of BYHW increases the protein levels of TGF-β and IL-10 in the brain of MCAO/reperfusion (I/R) rats. (**a and b**) The protein levels of TGF-β (a) and IL-10 (b), as measured by ELISA, in the brains obtained from sham-operated rats and I/R rats receiving vehicle, low-dose or high-dose powdered BYHW at 7 days after sham operation or I/R. Data were presented as mean ± SD (n = 3 in each group). ++, P < 0.01 and +++, P < 0.001 compared with I/R group; ##, P < 0.01 between groups treating with low-dose and high-dose powdered BYHW.

To further evaluate the protein levels of TNF-α and IL-6, ELISA was performed on cortical lysates obtained from sham-operated and I/R rats with and without BYHW treatment. Consistent with the results of immunohistochemistry, treatment with BYHW significantly attenuated I/R-induced elevation of these two proinflammatory cytokines (Figure 5A for TNF-α; Figure 5B for IL-6). Conversely, BYHW treatment increased the levels of anti-inflammatory cytokines including TGF-β (Figure 6A) and IL-10 (Figure 6B). Intriguingly, lower level of TNF-α and higher level of IL-10 were detected in the cerebral cortex of I/R rats treated with high dose of BYHW compared with I/R rats treated with low dose BYHW. These data suggest that post-I/R treatment with BYHW can improve the inflammatory profile in the cortex of I/R rats and the regulation of certain cytokines was dependent of the dose of BYHW.

### BYHW powdered product reduced lipid peroxidation in the cerebral cortex of rats

**Figure 7:**
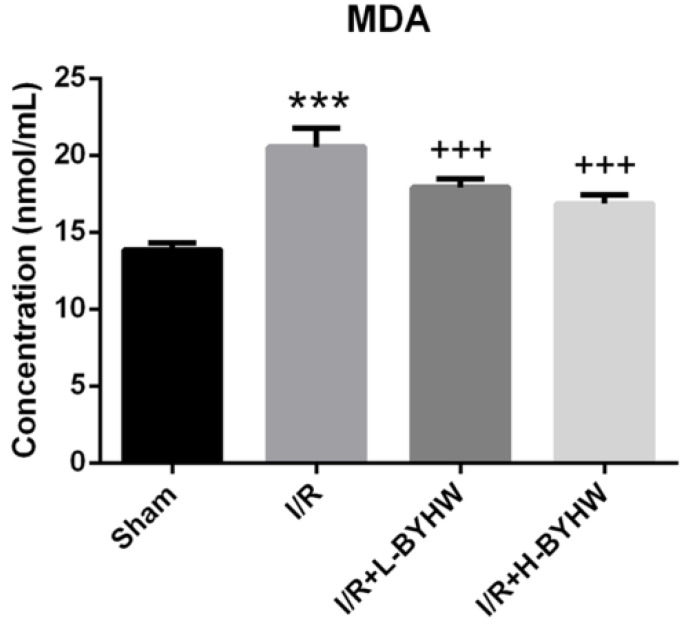
Powdered product of BYHW reduces lipid peroxidation in the cerebral cortex of MCAO/reperfusion (I/R) rats. (**a and b**) The level of malondialdehyde (MDA), as measured by ELISA, in the brains obtained from sham-operated rats and I/R rats receiving vehicle, low-dose or high-dose powdered BYHW at 7 days after sham operation or I/R. Data were presented as mean ± SD (n = 3 in each group). ***, P < 0.001 compared with sham-operated group; +++, P < 0.001 compared with I/R group.

Crosstalk between inflammation and oxidative stress has been extensively studied. Early release of reactive oxygen species and lipid peroxidation is suggested to have an important role in triggering neuroinflammation following ischemic stroke 14, 15, 16. To investigate the effects of different doses treatment of BYHW on oxidative stress, the level of MDA, a marker of lipid peroxidation, in the cortical lysates was examined by ELISA. The results showed that both low and high dose of BYHW treatments normalized I/R-induced increase of MDA level in rat brain and these two doses had comparable effects on the changes in MDA expression level (Figure 7). Taken together, these data suggest that the anti-inflammatory and anti-oxidative stress properties of BYHW contribute to its therapeutic activity and higher dose of BYHW may further improve the clinical outcome via modulation of inflammation.

## Discussion

Although it has been well-established that thrombolysis is the principal treatment for cerebral ischemia, the clinical outcome varies at a wide range among patients receiving this therapy and therefore cerebral ischemia remains one of the major causes of death and disability. Based on empirical knowledge, many TCMs including BYHW are considered to be beneficial for cerebral ischemic stroke for centuries. Here, we provide an evidence-based study that oral administrations of BYHW powdered product, after the onset of I/R, provide protection against I/R-induced cerebral infarction and neurobehavioral defects. Moreover, the dose-dependent therapeutic effect of BYHW is associated with its anti-inflammatory activity.

Traditionally, TCM decoction is prepared by mixing particular herbal components and boiling for hours. The procedure is time-consuming and difficult to control the quality of the final products. Nowadays, more and more powdered products of TCM are prescribed and used clinically including BYHW, but a lack of research supporting their use and the underlying mechanisms. In this study, BYHW powdered product is shown to have rescue effects on I/R-induced cerebral ischemic damage, which is in agreement with previous studies using water extract of BYHW or a combination of BYHW components 8, 9. While oral administrations of low dose BYHW powder after I/R induction significantly reduce the infarct area in parallel with a neurobehavioral improvement, it is striking to observe that, in high dose-treated I/R rats, infarct area is barely visible and corner test performance is close to that of sham-operated group at day 14 post-I/R. These findings suggest that BYHW powdered product can offer a more convenient option to manage I/R-induced neurobehavioral damage.

A cerebral inflammatory response plays an important role in the pathological mechanisms of ischemic stroke. For example, increased inflammatory reaction induces the production of free radicals, mitochondrial dysfunction, DNA damage, and lipid peroxidation 17. All of these pathological cascades are linked to neuronal death and further functional impairment 15. Although it remains controversial, elevation of proinflammatory cytokines (e.g., TNF-α, IL-6) to pathophysiological concentrations may induce neuronal and vascular dysfunction under brain ischemic condition 18, 19, 20. The present work shows that treatment with powdered BYHW significantly reverses the elevation of TNF-α and IL-6 in the brain of I/R rats. On the other hand, BYHW treatment is found to increase the protein level of anti-inflammatory cytokines including IL-10 and TGF-β in the brain. It has been demonstrated that treatment with IL-10-producing B-cells has been shown to reduce infarct size and peripheral and brain inflammation in an experimental stroke model 21. In addition, treatment with recombinant TGF-β or overexpression of TGF-β is shown to reduce inflammatory response and infarct volume in stroke animal models 22, 23. According to these findings, it is suggested that powdered BYHW has beneficial effects on ischemic stroke by providing anti-inflammatory environment.

In terms of the dose-dependent effects of powdered BYHW, significant lower level of TNF-α, but higher level of IL-10, is observed in the brain of high-dose BYHW-treated I/R rats compared with that of low-dose BYHW-treated I/R rats. These results suggest that the regulation of these two cytokines may contribute to the better therapeutic effect of high-dose BYHW. Furthermore, given that oxidative stress can regulate the expression of inflammatory cytokines by activating proinflammatory transcription factors 16, we also examine the effects of different doses treatment of BYHW on the cortical lipid peroxidation. Consistent with previous findings 5, BYHW treatment was shown to significantly inhibit oxidative stress. However, there are no differences in the level of MDA between low and high dose of BYHW. This suggests that dose-dependent effect of BYHW on the expression of TNF-α and IL-10 may be independent of the regulation by oxidative stress. Although it is unclear how BYHW treatment affects inflammatory signaling pathways, our present findings suggest that modulation of TNF-α and IL-10 levels can potentiate the effectiveness of powdered BYHW in cerebral ischemic stroke.

While the present study reveals that inflammatory modulation has an important role in I/R outcome under BYHW treatment, there are some limitations to our work that warrant further investigation. Inflammation has been shown to induce blood-brain barrier (BBB) injury. Higher BBB permeability is a pathological event of ischemic stroke and it is essential for the development of cerebral edema 24. HE staining showed that white cell infiltration and blood vessel rupture and bleeding are present in the brains of I/R rats, but these events are significantly attenuated in BYHW-treated I/R rats. These data suggest that BYHW treatment reduces not only inflammation but also the following vascular damage. In addition, neurotropic factors such as vascular endothelial growth factor (VEGF) may display different effect on blood vessels at different stages of I/R 9. Furthermore, although it has been well studied that over-expression of inflammatory cytokines by glial cells is critical for the pathogenesis of stroke, the correlation between peripheral inflammation and functional outcome of stroke remains elusive. Future study is needed to investigate whether the effects of BYHW on cerebral vascular system and peripheral inflammatory regulation are associated with the neuronal outcome of ischemia stroke.

To focus the research on the dose-dependent effect of BYHW treatment on brain inflammatory regulation and stroke outcome, male rats at fixed age were used to perform these experiments. Duo to lower stroke incidence observed in pre-menopausal women and poorer functional outcome observed in older individuals, many preclinical studies have been conducted to investigate the role of gender and age in the pathogenesis of stroke 6, 25. Accordingly, gonadal hormones and aging are found to affect the immune responses after stroke 6, 25. It will be interesting to examine whether the therapeutic efficacy of BYHW treatment is altered in a gender- or age-dependent manner in future study. While most studies including the present one examine the acute effects of BYHW on stroke, the effects of chronic treatment of BYHW are yet to be fully elucidated. Long-term effects of BYHW on suspected genes or target proteins related to inflammation and mitochondria homeostasis in ischemic stroke and peripheral artery diseases also warrant further research 9, 26, 27, 28.

In conclusion, these findings show that oral administrations of BYHW powdered product, after the onset of ischemia and reperfusion, improve neurological and functional outcome of experimental stroke. The protective mechanism of BYHW powdered product is associated with its anti-inflammatory actions. This study supports the idea that inflammatory modulation could be a therapeutic strategy for ischemic stroke.

## Figures and Tables

**Table 1:** The component of the powdered Bu Yang Huan Wu decoction (BYHW)

The following herbs yield an amount of dry extract		5 g
Huangqi (Radix Astragali seu Hedysari)	20 g	
Danggui (Radix Angelica sinensis)	1 g	
Chishao (Radix Paeoniae Rubra)	1 g	
Chuanxiong (Rhizoma Ligustici Chuanxiong)	0.5 g	
Honghua (Flos Carthami)	0.5 g	
Taoren (Semen Persicae)	0.5 g	
Dilong (Pheretima)	0.5 g	
**Corn starch**		4.2 g
**Powdered cellulose**		2.8 g

Each 12 g of the powdered BYHW contains the above components.
